# 1,1′-[4-(2-Methoxyphenyl)-2,6-dimethyl-1,4-dihydropyridine-3,5-diyl]diethanone

**DOI:** 10.1107/S1600536809042895

**Published:** 2009-10-28

**Authors:** B. Palakshi Reddy, V. Vijayakumar, J. Suresh, T. Narasimhamurthy, P. L. Nilantha Lakshman

**Affiliations:** a Organic Chemistry Division, School of Science and Humanities, VIT University, Vellore 632 014, India; b Department of Physics, The Madura College, Madurai 625 011, India; c Materials Research Centre, Indian Institute of Science, Bangalore 560 012, India; d Department of Food Science and Technology, Faculty of Agriculture, University of Ruhuna, Mapalana, Kamburupitiya 81100, Sri Lanka

## Abstract

In the title compound, C_18_H_21_NO_3_, the 1,4-dihydro­pyridine ring exhibits a flattened boat conformation. The methoxy­phenyl ring is nearly planar [r.m.s. deviation = 0.0723 (1) Å] and is perpendicular to the base of the boat [dihedral angle = 88.98 (4)°]. Inter­molecular N—H⋯O and C—H⋯O hydrogen bonds exist in the crystal structure.

## Related literature

For the biological importance of the 1,4-dihydro­pyridine ring, see: Gaudio *et al.* (1994 ▶); Böcker & Guengerich, (1986 ▶); Gordeev *et al.* (1996 ▶); Vo *et al.* (1995 ▶); Cooper *et al.* (1992 ▶). For hydrogen-bonding inter­actions, see: Bernstein *et al.* (1995 ▶).
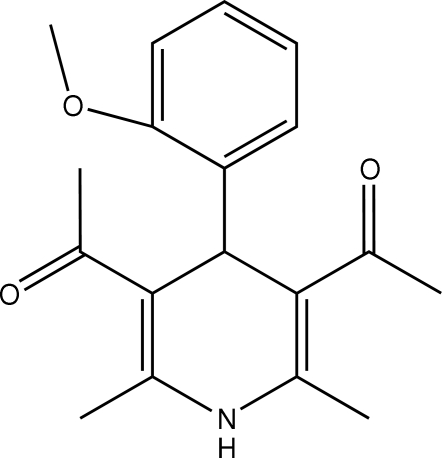


## Experimental

### 

#### Crystal data

C_18_H_21_NO_3_
*M* *_r_* = 299.36
Monoclinic, 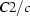

*a* = 26.5512 (6) Å
*b* = 7.5077 (1) Å
*c* = 17.0818 (3) Å
β = 114.904 (1)°
*V* = 3088.44 (10) Å^3^
*Z* = 8
Mo *K*α radiation
μ = 0.09 mm^−1^
*T* = 293 K
0.19 × 0.17 × 0.15 mm

#### Data collection

Bruker SMART APEX CCD diffractometer
Absorption correction: multi-scan (*SADABS*; Bruker, 1998 ▶) *T*_min_ = 0.984, *T*_max_ = 0.987
21137 measured reflections
4722 independent reflections
3203 reflections with *I* > 2σ(*I*)
*R*_int_ = 0.032

#### Refinement

*R*[*F* ^2^ > 2σ(*F* ^2^)] = 0.052
*wR*(*F* ^2^) = 0.154
*S* = 1.05
4722 reflections
208 parameters
H atoms treated by a mixture of independent and constrained refinement
Δρ_max_ = 0.26 e Å^−3^
Δρ_min_ = −0.23 e Å^−3^

### 

Data collection: *SMART* (Bruker, 2001 ▶); cell refinement: *SAINT* (Bruker, 2001 ▶); data reduction: *SAINT*; program(s) used to solve structure: *SHELXS97* (Sheldrick, 2008 ▶); program(s) used to refine structure: *SHELXL97* (Sheldrick, 2008 ▶); molecular graphics: *PLATON* (Spek, 2009 ▶); software used to prepare material for publication: *SHELXL97*.

## Supplementary Material

Crystal structure: contains datablocks global, I. DOI: 10.1107/S1600536809042895/ez2191sup1.cif

Structure factors: contains datablocks I. DOI: 10.1107/S1600536809042895/ez2191Isup2.hkl

Additional supplementary materials:  crystallographic information; 3D view; checkCIF report

## Figures and Tables

**Table 1:** Hydrogen-bond geometry (Å, °)

*D*—H⋯*A*	*D*—H	H⋯*A*	*D*⋯*A*	*D*—H⋯*A*
N1—H1⋯O1^i^	0.89 (2)	2.11 (2)	2.9749 (16)	163 (2)
C7—H7*B*⋯O1^i^	0.96	2.57	3.367 (2)	141
C15—H15⋯O3^ii^	0.93	2.50	3.381 (2)	158

Symmetry codes: (i) 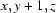; (ii) 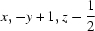.

**Fig. 1.**
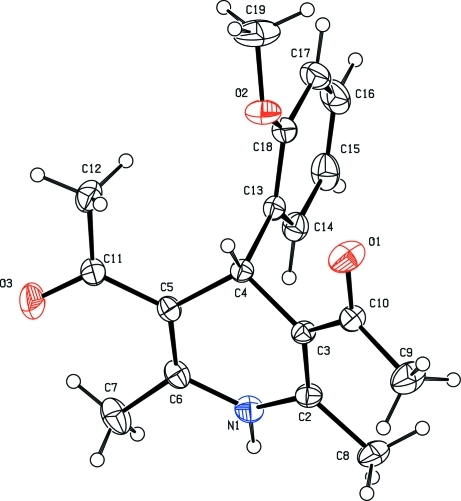
The molecular structure of title compound with atom numbering scheme and 30% probability displacement ellipsoids.

**Fig. 2.**
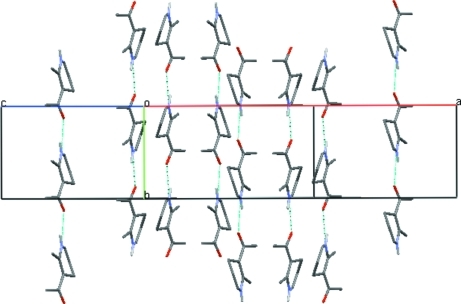
Partial packing view showing N—H···O bonding (dashed lines) generating a graph set motif of C(6). Atoms that do not take part in the hydrogen bonding have been omitted for clarity.

## supplementary crystallographic information

### Comment

1,4-dihydropyridines (1,4-DHPs) are biologically active compounds which include various vasodilator, antihypertensive, bronchodilator, heptaprotective, antitumor, antimutagenic, geroprotective and antidiabetic agents (Gaudio *et al.*, 1994). Nifedipine, Nitrendipine and Nimodipine *etc*., have found commercial utility as calcium channel blockers (Böcker & Guengerich, 1986; Gordeev *et al.*, 1996). For the treatment of congestive heart failure a number of DHP calcium antagonists have been introduced (Vo *et al.*, 1995). Some of DHPs have been introduced as a neuroprotectant and cognition enhancer. In addition, a number of DHPs with platelet antiaggregatory activity have also been discovered (Cooper *et al.*, 1992).

The configuration and conformation of the title compound, (I) and the atom numbering scheme are shown in the *ORTEP* drawing (Fig. 1). The 1,4-DHP ring exhibits a flattened boat conformation, with atoms N1 and C4 displaced by -0.165 (2) and -0.420 (2) Å, respectively, from the least-squares plane defined by the remaining four atoms of the DHP ring. The maximum deviation of these latter four atoms (C2/C3/C5/C6) from their mean plane is 0.015 (1) Å. The methoxy phenyl ring is nearly planar and is approximately perpendicular to the 1,4-DHP ring; the dihedral angle between the plane of the methoxyphenyl ring and the plane of the base of the boat(C2/C3/C5/C6) is 88.98 (4)°. Each carbonyl group is oriented in a synperiplanar (*cis*) or antiperiplanar (*trans*) conformation with respect to the adjacent C=C double bond of the 1,4-DHP ring. The observed torsion angles are C6/C5/C11/O3 [7.2 (3)°] and C2/C3/C10/O1 [174.3 (3)°], indicating *cis* and *trans* conformations, respectively. The carbonyl C10=O1 bond length of 1.226 (1)Å is somewhat longer than typical carbonyl bonds, possibly due to the involvement of atom O1 in an intermolecular N—H···O hydrogen bond.

In the crystal structure, intermolecular N—H···O, C—H···O hydrogen bonds (Table 1; Fig. 2) are observed, where the N—H···O bond generates a graph set motif of C(6) (Bernstein *et al.*, 1995), forming an infinite chain along the *b* axis.

### Experimental

3,5-diacetyl-2,6-dimethyl-1,4-dihydro-4-(2-methoxyphenyl)-pyridine was prepared according to the Hantzsch pyridine synthesis. 2-methoxybenzaldehyde (10 mmol), acetylacetone (20 mmol) and ammonium acetate (10 mmol) are taken in 1: 2: 1 mole ratio along with ethanol (25 ml) as a solvent in a RB-flask and refluxed in steam-bath until the color of the solution changes to reddish-orange (approximately 2 h). This mixture is kept under ice cold conditions to obtain the solid product, which is extracted using diethyl ether and acetone, then excess solvent was distilled off. The purity of the crude product was checked through TLC and recrystallized using an acetone and diethyl ether (1:1) solvent mixture. Yield 89.7%, M.P. 185–187 °C.

### Refinement

The N-bound H atom was located in a difference Fourier map and its positional parameters were refined. The H atoms were placed in calculated positions and allowed to ride on their carrier atoms with C—H = 0.93–0.98 Å.*U*_iso_ = 1.2*U*_eq_(C) for CH and *U*_iso_ = 1.5*U*_eq_(C) for CH_3_ groups.

### Crystal data

**Table:**

C_18_H_21_NO_3_	*F*(000) = 1280
*M**_r_* = 299.36	*D*_x_ = 1.288 Mg m^−^^3^
Monoclinic, *C*2/*c*	Mo *K*α radiation, λ = 0.71073 Å
Hall symbol: -C 2yc	Cell parameters from 2500 reflections
*a* = 26.5512 (6) Å	θ = 2–30°
*b* = 7.5077 (1) Å	µ = 0.09 mm^−^^1^
*c* = 17.0818 (3) Å	*T* = 293 K
β = 114.904 (1)°	Block, colourless
*V* = 3088.44 (10) Å^3^	0.19 × 0.17 × 0.15 mm
*Z* = 8	

### Data collection

**Table:**

Bruker SMART APEX CCD diffractometer	4722 independent reflections
Radiation source: fine-focus sealed tube	3203 reflections with *I* > 2σ(*I*)
graphite	*R*_int_ = 0.032
ω–scans	θ_max_ = 30.5°, θ_min_ = 1.7°
Absorption correction: multi-scan (*SADABS*; Bruker, 1998)	*h* = −37→37
*T*_min_ = 0.984, *T*_max_ = 0.987	*k* = −10→9
21137 measured reflections	*l* = −23→24

### Refinement

**Table:**

Refinement on *F*^2^	Primary atom site location: structure-invariant direct methods
Least-squares matrix: full	Secondary atom site location: difference Fourier map
*R*[*F*^2^ > 2σ(*F*^2^)] = 0.052	Hydrogen site location: inferred from neighbouring sites
*wR*(*F*^2^) = 0.154	H atoms treated by a mixture of independent and constrained refinement
*S* = 1.05	*w* = 1/[σ^2^(*F*_o_^2^) + (0.0675*P*)^2^ + 1.5117*P*] where *P* = (*F*_o_^2^ + 2*F*_c_^2^)/3
4722 reflections	(Δ/σ)_max_ < 0.001
208 parameters	Δρ_max_ = 0.26 e Å^−^^3^
0 restraints	Δρ_min_ = −0.23 e Å^−^^3^

### Special details

**Table:**

Geometry. All e.s.d.'s (except the e.s.d. in the dihedral angle between two l.s. planes) are estimated using the full covariance matrix. The cell e.s.d.'s are taken into account individually in the estimation of e.s.d.'s in distances, angles and torsion angles; correlations between e.s.d.'s in cell parameters are only used when they are defined by crystal symmetry. An approximate (isotropic) treatment of cell e.s.d.'s is used for estimating e.s.d.'s involving l.s. planes.
Refinement. Refinement of *F*^2^ against ALL reflections. The weighted *R*-factor *wR* and goodness of fit *S* are based on *F*^2^, conventional *R*-factors *R* are based on *F*, with *F* set to zero for negative *F*^2^. The threshold expression of *F*^2^ > σ(*F*^2^) is used only for calculating *R*-factors(gt) *etc*. and is not relevant to the choice of reflections for refinement. *R*-factors based on *F*^2^ are statistically about twice as large as those based on *F*, and *R*- factors based on ALL data will be even larger.

### Fractional atomic coordinates and isotropic or equivalent isotropic displacement parameters (Å^2^)

**Table:**

	*x*	*y*	*z*	*U*_iso_*/*U*_eq_	
H1	0.0342 (8)	0.604 (3)	0.1642 (13)	0.057 (6)*	
C2	0.01518 (6)	0.35160 (18)	0.13007 (9)	0.0323 (3)	
C3	0.03718 (6)	0.18412 (16)	0.14901 (8)	0.0281 (3)	
C4	0.09866 (6)	0.16431 (16)	0.20952 (8)	0.0269 (3)	
H4	0.1037	0.0490	0.2387	0.032*	
C5	0.11627 (6)	0.30983 (18)	0.27839 (8)	0.0303 (3)	
C6	0.09083 (7)	0.47109 (18)	0.25781 (9)	0.0347 (3)	
C7	0.10550 (9)	0.6384 (2)	0.31085 (12)	0.0535 (5)	
H7A	0.0815	0.6513	0.3396	0.080*	
H7B	0.1011	0.7391	0.2739	0.080*	
H7C	0.1434	0.6319	0.3530	0.080*	
C8	−0.03908 (7)	0.4093 (2)	0.05923 (11)	0.0460 (4)	
H8A	−0.0468	0.3383	0.0087	0.069*	
H8B	−0.0368	0.5324	0.0459	0.069*	
H8C	−0.0683	0.3940	0.0778	0.069*	
C9	−0.05561 (8)	0.0130 (2)	0.06598 (14)	0.0566 (5)	
H9A	−0.0653	0.0526	0.0080	0.085*	
H9B	−0.0726	0.0897	0.0929	0.085*	
H9C	−0.0686	−0.1067	0.0650	0.085*	
C10	0.00627 (6)	0.01838 (18)	0.11610 (9)	0.0336 (3)	
C11	0.16218 (7)	0.2748 (2)	0.36344 (10)	0.0389 (3)	
C12	0.19213 (7)	0.1016 (2)	0.37945 (10)	0.0468 (4)	
H12A	0.2249	0.1077	0.4328	0.070*	
H12B	0.2024	0.0768	0.3330	0.070*	
H12C	0.1684	0.0085	0.3829	0.070*	
C13	0.13442 (6)	0.16523 (18)	0.15858 (8)	0.0300 (3)	
C14	0.13751 (7)	0.3174 (2)	0.11442 (9)	0.0386 (3)	
H14	0.1178	0.4182	0.1167	0.046*	
C15	0.16912 (8)	0.3240 (3)	0.06698 (11)	0.0512 (4)	
H15	0.1706	0.4278	0.0383	0.061*	
C16	0.19800 (8)	0.1756 (3)	0.06301 (12)	0.0568 (5)	
H16	0.2191	0.1787	0.0313	0.068*	
C17	0.19603 (8)	0.0222 (3)	0.10549 (12)	0.0512 (4)	
H17	0.2156	−0.0779	0.1020	0.061*	
C18	0.16491 (6)	0.0155 (2)	0.15384 (10)	0.0376 (3)	
C19	0.20059 (10)	−0.2704 (3)	0.21025 (18)	0.0742 (7)	
H19A	0.1934	−0.3245	0.1556	0.111*	
H19B	0.1972	−0.3585	0.2485	0.111*	
H19C	0.2375	−0.2220	0.2348	0.111*	
N1	0.04566 (6)	0.49206 (16)	0.17923 (8)	0.0379 (3)	
O1	0.03123 (5)	−0.12395 (14)	0.13193 (9)	0.0566 (4)	
O2	0.16203 (5)	−0.13353 (15)	0.19817 (8)	0.0498 (3)	
O3	0.17653 (7)	0.3810 (2)	0.42269 (9)	0.0909 (6)	

### Atomic displacement parameters (Å^2^)

**Table:**

	*U*^11^	*U*^22^	*U*^33^	*U*^12^	*U*^13^	*U*^23^
C2	0.0344 (7)	0.0271 (6)	0.0361 (7)	0.0036 (5)	0.0156 (6)	0.0036 (5)
C3	0.0280 (7)	0.0241 (6)	0.0309 (6)	0.0009 (5)	0.0113 (5)	0.0010 (5)
C4	0.0284 (6)	0.0228 (5)	0.0282 (6)	0.0007 (5)	0.0106 (5)	0.0012 (4)
C5	0.0337 (7)	0.0288 (6)	0.0300 (6)	−0.0035 (5)	0.0151 (6)	−0.0026 (5)
C6	0.0457 (9)	0.0275 (6)	0.0341 (7)	−0.0048 (6)	0.0201 (7)	−0.0025 (5)
C7	0.0843 (14)	0.0299 (7)	0.0480 (9)	−0.0072 (8)	0.0295 (10)	−0.0090 (7)
C8	0.0389 (9)	0.0404 (8)	0.0526 (9)	0.0123 (7)	0.0131 (7)	0.0094 (7)
C9	0.0359 (9)	0.0457 (9)	0.0747 (13)	−0.0091 (7)	0.0100 (9)	−0.0078 (9)
C10	0.0327 (7)	0.0281 (6)	0.0371 (7)	−0.0018 (5)	0.0119 (6)	−0.0002 (5)
C11	0.0363 (8)	0.0468 (8)	0.0329 (7)	−0.0009 (7)	0.0139 (6)	−0.0057 (6)
C12	0.0450 (9)	0.0475 (9)	0.0361 (8)	0.0033 (7)	0.0056 (7)	0.0043 (7)
C13	0.0266 (7)	0.0340 (7)	0.0268 (6)	−0.0012 (5)	0.0085 (5)	−0.0027 (5)
C14	0.0392 (8)	0.0424 (8)	0.0336 (7)	−0.0014 (6)	0.0149 (6)	0.0030 (6)
C15	0.0555 (11)	0.0641 (11)	0.0377 (8)	−0.0147 (9)	0.0231 (8)	0.0014 (7)
C16	0.0535 (11)	0.0810 (14)	0.0478 (10)	−0.0131 (10)	0.0329 (9)	−0.0147 (9)
C17	0.0432 (10)	0.0629 (11)	0.0534 (10)	−0.0005 (8)	0.0262 (8)	−0.0162 (8)
C18	0.0326 (8)	0.0416 (8)	0.0358 (7)	0.0017 (6)	0.0117 (6)	−0.0070 (6)
C19	0.0605 (13)	0.0437 (10)	0.121 (2)	0.0168 (9)	0.0409 (14)	0.0013 (11)
N1	0.0481 (8)	0.0208 (5)	0.0426 (7)	0.0047 (5)	0.0170 (6)	0.0023 (5)
O1	0.0459 (7)	0.0242 (5)	0.0809 (9)	−0.0005 (5)	0.0083 (6)	−0.0021 (5)
O2	0.0494 (7)	0.0395 (6)	0.0653 (8)	0.0147 (5)	0.0288 (6)	0.0060 (5)
O3	0.0914 (12)	0.0885 (11)	0.0508 (8)	0.0342 (9)	−0.0110 (8)	−0.0358 (8)

### Geometric parameters (Å, °)

**Table:**

C2—C3	1.3667 (18)	C10—O1	1.2260 (17)
C2—N1	1.3783 (19)	C11—O3	1.2166 (19)
C2—C8	1.503 (2)	C11—C12	1.488 (2)
C3—C10	1.4662 (18)	C12—H12A	0.9600
C3—C4	1.5278 (18)	C12—H12B	0.9600
C4—C5	1.5274 (17)	C12—H12C	0.9600
C4—C13	1.5346 (19)	C13—C14	1.390 (2)
C4—H4	0.9800	C13—C18	1.407 (2)
C5—C6	1.358 (2)	C14—C15	1.392 (2)
C5—C11	1.475 (2)	C14—H14	0.9300
C6—N1	1.382 (2)	C15—C16	1.371 (3)
C6—C7	1.501 (2)	C15—H15	0.9300
C7—H7A	0.9600	C16—C17	1.374 (3)
C7—H7B	0.9600	C16—H16	0.9300
C7—H7C	0.9600	C17—C18	1.394 (2)
C8—H8A	0.9600	C17—H17	0.9300
C8—H8B	0.9600	C18—O2	1.3708 (19)
C8—H8C	0.9600	C19—O2	1.403 (2)
C9—C10	1.500 (2)	C19—H19A	0.9600
C9—H9A	0.9600	C19—H19B	0.9600
C9—H9B	0.9600	C19—H19C	0.9600
C9—H9C	0.9600	N1—H1	0.89 (2)
			
C3—C2—N1	118.48 (13)	C3—C10—C9	122.92 (13)
C3—C2—C8	128.68 (13)	O3—C11—C5	122.83 (15)
N1—C2—C8	112.82 (12)	O3—C11—C12	117.63 (15)
C2—C3—C10	125.14 (13)	C5—C11—C12	119.53 (13)
C2—C3—C4	118.56 (11)	C11—C12—H12A	109.5
C10—C3—C4	116.29 (11)	C11—C12—H12B	109.5
C5—C4—C3	110.16 (11)	H12A—C12—H12B	109.5
C5—C4—C13	111.83 (11)	C11—C12—H12C	109.5
C3—C4—C13	110.85 (10)	H12A—C12—H12C	109.5
C5—C4—H4	108.0	H12B—C12—H12C	109.5
C3—C4—H4	108.0	C14—C13—C18	117.30 (13)
C13—C4—H4	108.0	C14—C13—C4	120.21 (12)
C6—C5—C11	121.91 (13)	C18—C13—C4	122.49 (12)
C6—C5—C4	118.72 (12)	C13—C14—C15	122.19 (16)
C11—C5—C4	119.29 (12)	C13—C14—H14	118.9
C5—C6—N1	119.11 (12)	C15—C14—H14	118.9
C5—C6—C7	127.97 (15)	C16—C15—C14	119.16 (17)
N1—C6—C7	112.92 (13)	C16—C15—H15	120.4
C6—C7—H7A	109.5	C14—C15—H15	120.4
C6—C7—H7B	109.5	C15—C16—C17	120.58 (16)
H7A—C7—H7B	109.5	C15—C16—H16	119.7
C6—C7—H7C	109.5	C17—C16—H16	119.7
H7A—C7—H7C	109.5	C16—C17—C18	120.49 (16)
H7B—C7—H7C	109.5	C16—C17—H17	119.8
C2—C8—H8A	109.5	C18—C17—H17	119.8
C2—C8—H8B	109.5	O2—C18—C17	122.79 (14)
H8A—C8—H8B	109.5	O2—C18—C13	116.94 (13)
C2—C8—H8C	109.5	C17—C18—C13	120.26 (15)
H8A—C8—H8C	109.5	O2—C19—H19A	109.5
H8B—C8—H8C	109.5	O2—C19—H19B	109.5
C10—C9—H9A	109.5	H19A—C19—H19B	109.5
C10—C9—H9B	109.5	O2—C19—H19C	109.5
H9A—C9—H9B	109.5	H19A—C19—H19C	109.5
C10—C9—H9C	109.5	H19B—C19—H19C	109.5
H9A—C9—H9C	109.5	C2—N1—C6	123.47 (12)
H9B—C9—H9C	109.5	C2—N1—H1	120.1 (13)
O1—C10—C3	119.46 (13)	C6—N1—H1	116.0 (13)
O1—C10—C9	117.58 (13)	C18—O2—C19	118.17 (15)
			
N1—C2—C3—C10	166.44 (14)	C4—C5—C11—C12	2.6 (2)
C8—C2—C3—C10	−15.2 (2)	C5—C4—C13—C14	−57.93 (16)
N1—C2—C3—C4	−13.2 (2)	C3—C4—C13—C14	65.43 (16)
C8—C2—C3—C4	165.14 (14)	C5—C4—C13—C18	122.36 (14)
C2—C3—C4—C5	34.57 (16)	C3—C4—C13—C18	−114.29 (14)
C10—C3—C4—C5	−145.08 (12)	C18—C13—C14—C15	0.1 (2)
C2—C3—C4—C13	−89.74 (15)	C4—C13—C14—C15	−179.60 (14)
C10—C3—C4—C13	90.61 (14)	C13—C14—C15—C16	0.4 (3)
C3—C4—C5—C6	−31.67 (17)	C14—C15—C16—C17	−0.2 (3)
C13—C4—C5—C6	92.08 (15)	C15—C16—C17—C18	−0.5 (3)
C3—C4—C5—C11	151.55 (12)	C16—C17—C18—O2	−179.28 (16)
C13—C4—C5—C11	−84.71 (15)	C16—C17—C18—C13	1.0 (3)
C11—C5—C6—N1	−175.72 (13)	C14—C13—C18—O2	179.44 (13)
C4—C5—C6—N1	7.6 (2)	C4—C13—C18—O2	−0.8 (2)
C11—C5—C6—C7	4.3 (2)	C14—C13—C18—C17	−0.8 (2)
C4—C5—C6—C7	−172.37 (15)	C4—C13—C18—C17	178.91 (14)
C2—C3—C10—O1	174.28 (15)	C3—C2—N1—C6	−15.2 (2)
C4—C3—C10—O1	−6.1 (2)	C8—C2—N1—C6	166.19 (14)
C2—C3—C10—C9	−8.1 (2)	C5—C6—N1—C2	18.3 (2)
C4—C3—C10—C9	171.54 (15)	C7—C6—N1—C2	−161.78 (15)
C6—C5—C11—O3	7.2 (3)	C17—C18—O2—C19	13.7 (2)
C4—C5—C11—O3	−176.16 (17)	C13—C18—O2—C19	−166.57 (17)
C6—C5—C11—C12	−174.06 (15)		

### Hydrogen-bond geometry (Å, °)

**Table:**

*D*—H···*A*	*D*—H	H···*A*	*D*···*A*	*D*—H···*A*
N1—H1···O1^i^	0.89 (2)	2.11 (2)	2.9749 (16)	163 (2)
C7—H7B···O1^i^	0.96	2.57	3.367 (2)	141
C15—H15···O3^ii^	0.93	2.50	3.381 (2)	158

Symmetry codes: (i) *x*, *y*+1, *z*; (ii) *x*, −*y*+1, *z*−1/2.
